# Learners without borders

**DOI:** 10.1017/gmh.2014.6

**Published:** 2014-09-15

**Authors:** Helen Verdeli

We are launching the Teaching and Learning (T&L) section of *Global Mental Health* (*GMH*) in a spirit of celebration grounded in deep awareness of responsibility. In concert with the Journal's mission of moving from ***making the case*** to ***implementing*** GMH (Belkin, 2014), the T&L section's mandate is the public health need for effective and widely accessible mental health teaching and learning methods, tools, metrics, networks, and communities. We welcome your contributions so we can tackle what historically has been the greatest contributor to mental health treatment gaps: lack of an adequately (in numbers and capacity) trained mental health workforce (Scheffler & World Health Organization, 2011). This priority from the field is echoed in the research world: during a global Delphi workshop to identify mental health research priorities in low- and middle-income countries (LMICs), training of community health workers in evidence-based care was rated as a top priority (Collins *et al.,* 2011). And the landmark Mental Health Action Plan by the World Health Assembly articulated as a key actionable target the development of knowledge and skills in mental health services grounded in scientific evidence, contextual understanding, and commitment to human rights (World Health Organization, 2013).

With teaching and learning of the mental health workforce as its central axis, the T&L priority areas include:

• Innovations in the development of feasible, contextually informed, effective, and cost-effective training (workshops, manuals, and supervision) with members of the mental healthcare workforce, both specialists and non-specialists.
• Provider and supervisor skill competency assessment, life-long learning, and skill reinforcement programs, ongoing program quality monitoring and improvement procedures, provider burden reduction and burnout prevention, user-friendly clinical management and decision-support m- and e-tools, organizational/policy factors supporting ongoing skill development, wellbeing of trainers and trainees, etc.
• Increase of community engagement and advocacy, family and person involvement in treatment, community mental health literacy, stigma reduction strategies and community impact of interventions on mental health knowledge and attitudes, etc.
• Effective strategies to maximize the reach of state-of-the-art mental health skills to academics, practitioners, and trainees globally; ethics in training; models for sustainable capacity-building and brain-drain prevention strategies; development of new models of learning communities, collaboratives, and global classrooms; managing learning stimulus ‘overload’ in the e-age, etc.

We hope that the T&L platform will set in motion a number of dialogues, discovery paths, and collaborations stemming from research projects, training programs, services, and policy/advocacy initiatives. Thus, teachers and learners in this section can not only be clinicians, and researchers, but also primary care personnel, development/aid organization workers, managers, policy makers, religious leaders, community members, and most critically, families and patients themselves. The common denominator is a focus on under-resourced systems in places where communities struggle with chronic adversity, toxic stress, and social exclusion on the one hand, and low availability of and access to mental health care for those who need it on the other. The problems that GMH tackles are everybody's problems.

However, the solutions are everybody's solutions as well. What 15 years ago was a radical proposition in mental health care is now an axiom: the number and globally capacity of mental health workforce will increase only by engaging and training persons with non-mental health background. A fast growing body of research showed us that after supervised training, lay counselors could safely deliver culturally adapted, and evidence-based skills to their communities in sub-Saharan Africa, primary care clinics in India and Chile, or homes in Pakistan (Chowdhary *et al.* 2014). These *task-shifting* strategies (or *task-sharing* when there is an available team to share the tasks) (Kakuma *et al.* 2011), have been posing unique opportunities but also unprecedented challenges for knowledge-sharing. The community health workers, who are frequently responsible for a number of health tasks and have no mental health background, need to develop mental health skillsets that are broad and versatile but also manageable, clearly articulated, and competently provided. This is a tall order.

If the trajectory continues, the next decade will bring a proliferation of tools for innovative, user-friendly models of training workshops, manuals, and supervision on evidence-based elements of care (assessment, low- and high-intensity psychosocial interventions, pharmacological interventions, etc.). It will also hopefully see growth in learning-driven implementation strategies, such as Quality Improvement, that empower local decision-makers and stakeholders to be more effective implementers.

In its most dynamic and comprehensive initiative to date, the WHO Mental Health and Substance Abuse department launched the mhGAP Intervention Guide (World Health Organization, 2010), by harnessing global consensus to build technical tools for the management of key mental, neurological, and substance use disorders for non-specialists. In the context of the Guide, partners from academia, NGOs, government, and research institutions have formed global collaborations to build these training tools (e.g., training modules for the mhGAP guide interview). The tools are accessible through widely disseminated media such as internet-based manuals or demonstration tapes on Youtube. Your work will extend and perhaps redirect these partnerships and consensus-building around these globally aligned goals.

Although a lot of attention is historically given to the quality of evidence that informs the selection of elements of care – the selection of a specific psychotherapy for example – there is significantly less on the quality of evidence of the training tools and processes themselves: did the training workshop increase knowledge in the domains targeted? Was there exploration of the cultural relevance of the training material? Was there a systematic process for its contextual adaptation? One point, however, of broad agreement, at least in theory, is the critical role of supervision for skill-building. There is a lot to be learned about facilitating and ‘culture-changing’ factors on the system and policy level, so that protected time for supervision becomes part of the workload and supervisors are not merely ‘compliance monitors’ but rather sources of support, knowledge, and improvement agents of providers' quality of work and life. The field is also looking for new ways of providing easily accessible stakeholder-driven feedback on training tools and methods. Finally, sustainable and scalable ways of capacity-building within academic institutions, which prepare the next generation of trainers, supervisors, and providers, as well as policies to prevent brain drain, are areas of great relevance to T&L. These topics are relevant to high- and low-income areas alike, and while we look to highlight solutions for low-resourced settings, we also look for contributions from any setting that inform us on shared challenges of broad applicability and interest.

GMH training poses increasing and novel demands. To meaningfully engage and share knowledge with adult learners from vastly different professional, cultural, geographical, and economic backgrounds, we need to be ‘multilingual’ discipline-wise. We need guidance from fields like adult education and learning, information management sciences, public health, social sciences, and therapeutics, amongst others.

A learning domain we cover in this section involves the task-shifting aspects of care to the person and family, usually termed patient and family engagement and psychoeducation. In recent years, most gains in the management of chronic diseases were achieved by self-monitoring (Pearson *et al.* 2007), and patient-initiated prevention/prophylaxis and treatment. Models of education of person and family about course of illness and recovery, available treatments and resources, would help the transition from compliance to alliance to empowerment and self-determination. The development of versatile, culturally meaningful m- and e- tools for self-initiated assessment and treatment is already, and will become an even more significant source of learning and change for person and family. We should note that the jury is still out about long-term efficacy of technology-only-assisted psychoeducation.

The demands of distance-learning and the need for training large numbers of learners are giving technology a leading role. In addition to geography, other concerns such as availability of experts, safety, health, climate, gender-related concerns, time commitments, cost, etc. make internet-based learning a realistic educational option for a large number of learners. Mental health internet-based models offer scalable alternatives to widely used in-person cascade models of ‘training-of-trainers’ but also increase access to specialized training when needed. The development and testing of such a model on a large scale is expected to greatly inform the field (Fairburn & Patel, 2014). The exponentially growing world of digital, online, and mobile-assisted platforms not only offers the convenience of choice between synchronous and asynchronous learning, but it also makes communication possible among new communities and facilitates knowledge exchange between all levels of consumers - from trainees to providers to service-users themselves. Media such as Second Life give the trainee space to safely practice freshly acquired skills before working with patients (Barnett, 2011). This new culture brings its own ethical, clinical, legal, and technical challenges that need to be documented. We should also remember that excellent Teaching and learning can take place with low technology but with the use of active learning principles, good old role-plays, quizzes during and after training, and use of simple checklists as follow-up reminders. Innovation means new solutions to old problems, but perhaps also old solutions to new problems. We are after all looking for good solutions; and in this spirit, we warmly welcome you to participate and send us your comments to globalmentalhealth@cambridge.org.
